# Physiological and Metabolic Mechanisms of *Penicillium sclerotigenum*-Induced Postharvest Rot in Lichuan Yam (*Dioscorea polystachya* Turcz.)

**DOI:** 10.3390/jof12030225

**Published:** 2026-03-19

**Authors:** Xiaoxiao Sun, Zhichao Wang, Yun Huang, Liya Zhang, Yuchang Zhu, Dazhai Zhou, Kun Xiong, Yan Qin, Kelin Li

**Affiliations:** 1 Hubei Provincial Key Laboratory of Biological Resource Conservation and Utilization, Hubei Minzu University, Enshi 445000, China; 202330401@hbmzu.edu.cn (X.S.);; 2 Hubei Provincial Key Laboratory of Selenium Resource Research and Biological Application, Hubei Minzu University, Enshi 445000, China; 3 School of Biological and Food Engineering, Hubei Minzu University, Enshi 445000, China; 4 College of Forestry and Horticulture, Hubei Minzu University, Enshi 445000, China; 5 Enshi Tujia and Miao Autonomous Prefecture Public Inspection and Testing Center, Enshi 445000, China

**Keywords:** yam, *Penicillium sclerotigenum*, decay, physiological mechanism, non-targeted metabolomics

## Abstract

Postharvest decay results in substantial losses during yam storage. This study isolated microorganisms from decayed Lichuan yams and investigated deterioration mechanisms using physiological assays and UPLC-MS/MS. Among six isolates, *Penicillium sclerotigenum* was identified as the primary pathogen. Infection disrupted water-retaining structures, leading to increased weight loss and reduced water activity. It also disrupted carbon-nitrogen metabolism, leading to fluctuations in starch, sugar, and protein content. Although host defense responses were activated via phenolic accumulation and the upregulation of peroxidase (POD) and polyphenol oxidase (PPO) activities, sustained infection resulted in severe membrane lipid peroxidation. Metabolomics revealed alterations in sugars, organic acids, and secondary metabolites, with the specific enrichment of sugar and amino acid pathways. Thus, *P. sclerotigenum* remodels yam energy metabolism and defense responses. This study clarifies the physiological and metabolic mechanisms underlying this fungal rot, providing a theoretical foundation for the development of preventive control strategies.

## 1. Introduction

Yam (*Dioscorea polystachya* Turcz.) is an important food and medicinal crop in China. Rich in starch, protein, polysaccharides, and various bioactive compounds, it possesses high nutritional and economic value [1,2,3]. However, during harvesting, transportation, storage, and marketing, yams are prone to skin damage and breakage. The resulting wounds are susceptible to microbial infection, leading to severe rot and deterioration in tuber quality. This phenomenon causes significant economic losses for local farmers and has become a critical constraint on industry development [4]. Therefore, research on yam postharvest diseases is imperative.

Postharvest rot is predominantly caused by fungi, with *Penicillium* species being particularly prevalent [5,6]. In Nigeria, researchers isolated *Aspergillus*, *Alternaria*, and *Penicillium* fungi from rotted yams [4,7,8]. In Japan, three *Penicillium* species—*P. albocoremium*, *P. polonicum*, and *P. sclerotigenum*—have been identified as pathogens causing green mold disease in yams [9]. However, current research on yam postharvest diseases primarily focuses on disease surveys, preliminary pathogen identification, and evaluation of preservation techniques, with relatively limited analyses of pathogenic species and their decay mechanisms.

During the initial stages of microbial infection, the host shows significant changes in moisture content, nutrient composition, enzyme activity, and indicators of oxidative stress. In tomatoes infected by *Botrytis cinerea*, plant defense proteins are upregulated early, while pathogen-induced enzymatic degradation reduces total protein content [10]. *Venturia inaequalis* infection of apples induces phenolic metabolism pathways in the peel, leading to increased phenolic compound levels at the infection site [11]. During the early stages of *Ceratocystis fimbriata* infection of sweet potatoes, soluble sugars and antioxidant enzyme activities undergo significant changes [12,13]. Thus, alterations in these indicators can systematically reveal the physiological and metabolic dynamics of host–pathogen interactions during infection.

Metabolomics technology helps elucidate the mechanisms underlying pathogen–host interactions. Its application facilitates research into various pathogenic mechanisms, such as potato dry rot, citrus anthracnose, and gray mold in berry fruit [14,15,16]. In studies of *Fusarium sambucinum* infection in potatoes, metabolomics not only successfully identified differentially expressed metabolites (DEMs) post-infection but also provided evidence for biomarker screening and validation [16]. Currently, systematic integrated analyses using metabolomics and related technologies to elucidate the mechanisms by which yams respond to pathogen infection at the metabolic network level remain scarce, with relevant research still in its exploratory phase.

Therefore, this study utilized Lichuan yam exhibiting typical rot symptoms as material, aiming to isolate and identify the dominant pathogenic microorganisms causing its postharvest decay, and to clarify their pathogenicity through in vitro inoculation experiments. Subsequently, we comprehensively employed physiological and biochemical methods to dynamically analyze the effects of pathogen infection on the yams’ water status, major nutritional components, defense substances, enzyme activities, and oxidative stress indicators. Finally, non-targeted metabolomics technology was employed to decipher the host’s holistic metabolic shifts and key pathway perturbations during critical infection stages. Through these investigations, we systematically elucidated the physiological and metabolic mechanisms by which dominant pathogens induce postharvest decay in yams, aiming to provide a theoretical foundation and data support for developing eco-friendly, efficient postharvest disease control technologies.

## 2. Materials and Methods

### 2.1. Materials

Typical rotted Lichuan yam samples were collected from a local farmers’ market in Tuanbao Town, Lichuan City, Hubei Province, China. After collection, samples were immediately placed in sterile foam boxes and transported to the laboratory within 2 h. Healthy, undamaged yams used for pathogenicity assays and subsequent experiments were sourced from a production base in the same region and delivered within 24 h of harvest. All samples were stored at 4 °C and processed within 24 h after receipt in the laboratory.

### 2.2. Isolation, Purification, and Identification of Microorganisms from Decayed Tissue

#### 2.2.1. Sample Preparation and Microbial Isolation and Purification

Tissue from the diseased-healthy interface was first rinsed three times with sterile water, then disinfected with 75% (*v*/*v*) ethanol for 30 s, and finally rinsed twice with sterile water. A 1 cm^3^ tissue sample was placed in 9 mL sterile physiological saline and vortexed for 2 min to prepare a microbial suspension. A tenfold serial dilution was performed. Then 100 μL of each dilution was taken and plated onto Beef Peptone Medium (BPM, incubated at 37 °C) and Potato Dextrose Agar (PDA, incubated at 28 °C) for 48–72 h, with three replicates per dilution level [7]. Morphologically distinct single colonies were isolated, streak-purified 3–5 times until uniformity was observed under microscopy, and stored on slant culture medium at 4 °C.

#### 2.2.2. Microbial Identification

Preserved strains were transferred to fresh culture plates and incubated at the optimal temperature for 48–72 h, and colony characteristics were recorded. For bacteria, bacterial smears were prepared by suspending a small number of colonies in sterile water. For fungi, fungal spores or hyphae were collected using the tape method. Their morphological structures were observed and documented under an optical microscope (model E200; Nikon, Tokyo, Japan) [17].

Genomic DNA from the test strains was extracted using the TGuide S96 Magnetic Bead-Based Soil/Fecal Genomic DNA Extraction Kit according to the manufacturer’s protocol. Primer sequences used in the experiment are detailed in Table S1. The PCR amplification reaction mixture (10 μL) consisted of: 2.5–4 ng genomic DNA, 0.3 μL VnF (10 μM), 0.3 μL VnR (10 μM), 5 μL KOD FX Neo Buffer, 2 μL dNTP (2 mM each), and 0.2 μL KOD FX Neo, and ddH_2_O was added to a final volume of 10 μL. The PCR amplification program was as follows: 95 °C predenaturation for 5 min, 95 °C denaturation for 30 s, 50 °C annealing for 30 s, 72 °C extension for 40 s, repeated for 25 cycles, followed by a 7 min extension at 72 °C and storage at 4 °C. The amplified products were sent to Beijing Tsingke Biotech Co., Ltd. (Wuhan, China), for sequencing. Sequence data were imported into the NCBI database for BLAST homology comparison (https://blast.ncbi.nlm.nih.gov/Blast.cgi, accessed on 10 July 2025). Phylogenetic trees were constructed using MEGA11 software. Strain species were determined based on the morphology of the putrefactive microbial [18].

### 2.3. Pathogenicity Assays for Major Putrefactive Microorganisms

The identified pure bacterial strains were inoculated into their respective media and incubated at the optimal temperature for 3 to 5 days. Bacterial cells and spores were collected with sterile water, and the concentration of each microbial suspension was then adjusted to 1 × 10^7^ CFU/mL using a hemocytometer (Chongqing Xinglan Technology Co., Ltd., Chongqing, China). Meanwhile, excluding the control group, the remaining test strains were resuspended in equal volumes, and the mixed-inoculation treatment group was then set up.

Sterilized, healthy, uniform yam segments (soaked in 1% (*v*/*v*) sodium hypochlorite solution for 3 min, rinsed three times with sterile water, and air-dried) were cut into 5 cm long cylindrical segments. A hole (5 mm diameter × 2 mm depth) was punched at the center of each cross-section. Each hole was inoculated with 5 μL of microbial suspension, with sterile water serving as the control. Six replicates were set up per group. After inoculation, the yam segments were placed in high-humidity sterile culture boxes and incubated at 28 °C for 5 days [15]. Disease development was observed and recorded daily for 5 consecutive days. The primary pathogen with the strongest pathogenicity was identified by comparing lesion expansion rates and severity.

### 2.4. Preparation of Pathogen Infection Dynamics Samples

Based on pathogenicity test results, the identified primary pathogenic fungus (*P. sclerotigenum*) was prepared as a spore suspension (1 × 10^7^ CFU/mL) as described in Section 2.3 and inoculated onto healthy yam segments (PI group). The control group was treated with an equal volume of sterile water. All treatments were incubated at 28 °C for 5 days and sampled daily: a 1 cm thick tissue section was cut with the inoculation point as the center, and samples were collected using a 2 cm diameter punch from this section. Samples were immediately flash-frozen in liquid nitrogen and stored at −80 °C for subsequent use.

### 2.5. Physiological and Biochemical Parameter Measurements

#### 2.5.1. Determination of Weight Loss Ratio and Water Activity

The percentage of weight loss of yams was calculated using Formula (1). In this formula, m_0_ denotes the initial weight (g) of each yam segment before inoculation, and m_t_ denotes the weight (g) of the yam segment at each sampling time point.

(1) $$Weight\;loss\;ratio\;\left(\%\right)=\frac{m_{0}-m_{t}}{m_{0}}$$

Water activity was measured using a water activity meter. A total of 1.00 g of yam tissue was weighed, placed in the dedicated sample dish of the water activity meter, and sealed immediately. The instrument was allowed to equilibrate automatically at 25 °C, and readings were taken until two consecutive values differed by less than 0.005; the final stable value was recorded.

#### 2.5.2. Determination of Soluble Sugars, Starch, and Protein Content

The contents of soluble sugars and starch were determined using the phenol-sulfuric acid method [19]. A standard curve was prepared using glucose as the standard substance (y = 0.0085x − 0.0012, R^2^ = 0.9991). A total of 1.0 g of the sample was weighed and extracted with 80% (*v*/*v*) ethanol solution in a boiling water bath for 30 min, and the supernatant was used for soluble sugar determination. The residue was hydrolyzed with perchloric acid, diluted to volume, and used for starch determination. The absorbance was measured at 490 nm. The results were expressed as percentage content (%).

Protein content was determined using the Coomassie Brilliant Blue G-250 method [20]. A standard curve was prepared using bovine serum albumin as the standard (y = 0.0050x + 0.0075, R^2^ = 0.9992). The measurement wavelength was 595 nm. Results were expressed as g kg^−1^.

#### 2.5.3. Determination of Total Phenolic and Malondialdehyde (MDA) Contents and Polyphenol Oxidase (PPO) and Peroxidase (POD) Activity

Total phenolic content was determined using the Folin–Ciocalteu method [21]. A standard curve was prepared using gallic acid as the standard (y = 0.0377x − 0.0177, R^2^ = 0.9992). The measurement wavelength was 760 nm. Results were expressed as g kg^−1^.

MDA content was determined using the thiobarbituric acid method [22]. MDA content in yams was calculated using Formula (2) and expressed in mmol kg^−1^. In this formula, V represents the total volume of the extract (mL), and m represents the fresh weight of the sample (g).

(2) $$MDA\;content({mmol\;kg}^{-1})=\frac{[6.45(A_{532}-A_{600})-0.56A_{450}]\times V}{m}$$

The phenol method was used to determine PPO activity at 420 nm [23]. The guaiacol method was employed to measure POD activity at a wavelength of 470 nm [24]. Crude enzyme solutions were extracted with pre-chilled phosphate buffer (0.1 mol/L, pH 7.8). Enzyme activity units (U) were defined as the amount of enzyme required to increase absorbance by 0.01 per minute. Enzyme activity in yams was calculated using Formula (3) and expressed in U. In this formula, ΔA represents the change in absorbance per minute, V_t_ denotes the total volume of crude enzyme solution (mL), m is the fresh weight of the sample (g), V_s_ is the volume of enzyme solution used for measurement (mL), and t is the reaction time (minutes).

(3) $$Enzyme\;activity\;(U)=\frac{\Delta A\times V_{t}}{0.01\times m\times V_{s}}$$

### 2.6. Non-Targeted Metabolomics Analysis

Based on the trends in physiological indicators, day 3 post-pathogen infection was identified as the critical time point. Frozen samples from the PI group and control group collected on day 3, preserved as described in Section 2.5, were sent to Wuhan Maiwei Biotechnology Co., Ltd. (Wuhan, China), for non-targeted metabolomics analysis using UPLC-MS/MS technology. Specific UPLC-MS/MS conditions were listed in Tables S2 and S3.

### 2.7. Data Processing and Statistical Analysis

All physiological and biochemical experiments included three independent biological replicates. Data were presented as mean ± standard deviation. Statistical analysis was performed using SPSS 26.0 software. Independent samples *t*-tests were used to identify significant differences between the PI group and the control group at the same time point (*: *p* < 0.05, **: *p* < 0.01). Visualization charts were generated using Origin Pro 2024 software and the Metware Cloud online platform (https://cloud.metware.cn/, accessed on 8 January 2026).

## 3. Results

### 3.1. Microbial Isolation and Identification of Primary Pathogens

**Figure 1:**
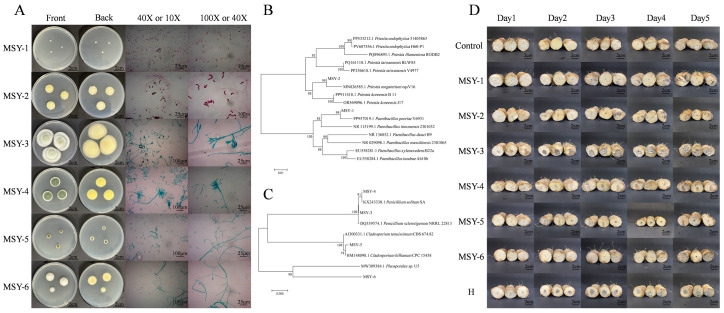
Microbial isolation and identification of primary pathogens. (**A**) Morphological characteristics of microorganisms; (**B**) Phylogenetic tree constructed from bacterial 16S rDNA sequences; (**C**) Phylogenetic tree constructed from fungal ITS rDNA sequences; (**D**) Decay results after inoculating different strains onto healthy yam cross-sections.

From naturally decaying yam tissue, we isolated and purified six microbial strains (designated MSY-1 to MSY-6). Combining morphological characteristics (Figure 1A) with phylogenetic tree analysis results (Figure 1B,C), we confirmed the taxonomic classification of these six strains. MSY-1 and MSY-2 were bacteria, identified as *Paenibacillus peoriae* and *Bacillus megaterium*, respectively. MSY-3 to MSY-6 were filamentous fungi, identified as *P.* *sclerotigenum* (MSY-3), *P.* *solitum* (MSY-4), *Cladosporium hillianum* (MSY-5), and *Pleosporales* sp. (MSY-6). Pathogenicity was further assessed through in vitro inoculation tests (Figure 1D). Results showed that healthy yams inoculated with *P. sclerotigenum* exhibited significantly faster lesion expansion rates and larger final rot areas compared to treatments with other single strains or mixed fungal suspensions. Thus, this study identified *P. sclerotigenum* as the primary pathogen causing postharvest rot in Lichuan yams.

### 3.2. Effects of P. sclerotigenum Infection on Yam Water Metabolism

**Figure 2:**
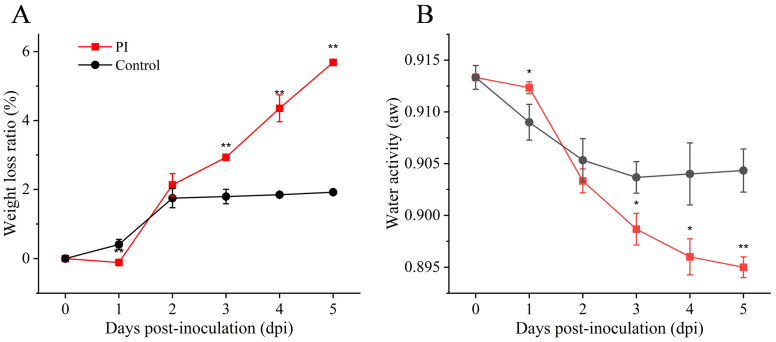
Effects of *P. sclerotigenum* infection on water metabolism in yams. (**A**) Weight loss ratio; (**B**) Water activity. All values are expressed as mean ± standard deviation (*n* = 3). Analysis performed using Student’s *t*-test, *: *p* < 0.05, **: *p* < 0.01.

This study found that pathogen infection significantly impaired the water retention capacity of yams (Figure 2A). Starting from the second day post-inoculation, the weight loss rate in the *P. sclerotigenum*-inoculated treatment group rapidly increased, reaching approximately 6.0% by day 5. In contrast, the control group maintained a consistently low weight loss ratio of approximately 1.8% throughout the observation period. Similarly, the water activity of *P. sclerotigenum*-infected yam tissues exhibited a continuous downward trend (Figure 2B). By day 5, the water activity in the PI group was significantly lower than that in the control group. These results indicate that *P. sclerotigenum* infection severely disrupts the water-retaining structures within yam tissues.

### 3.3. Effects of P. sclerotigenum Infection on Yam Carbon and Nitrogen Reserve Metabolism

**Figure 3:**
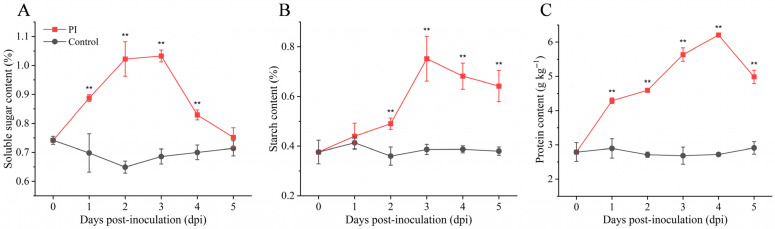
Effects of *P. sclerotigenum* infection on carbon and nitrogen reserve metabolism in yams. (**A**) Soluble sugar content; (**B**) Starch content; (**C**) Protein content. All values are expressed as mean ± standard deviation (*n* = 3). Analysis performed using Student’s *t*-test, **: *p* < 0.01.

As shown in Figure 3, *P. sclerotigenum* infection persistently disrupted the carbon-nitrogen metabolic balance in yams. Soluble sugar content rapidly accumulated during the first 3 days post-infection, peaking on day 3 at a level significantly higher than that of the control group, followed by a gradual decline (Figure 3A). Starch content exhibited a similar “rise-then-fall” pattern, also accumulating to its maximum level on day 3 (Figure 3B). Concurrently, total protein content rose significantly from the onset of infection, peaking on day 4 before declining (Figure 3C). In contrast, all metabolic indicators in the control group remained relatively stable throughout the storage period.

### 3.4. Defense Responses and Oxidative Damage Induced by P. sclerotigenum Infection

**Figure 4:**
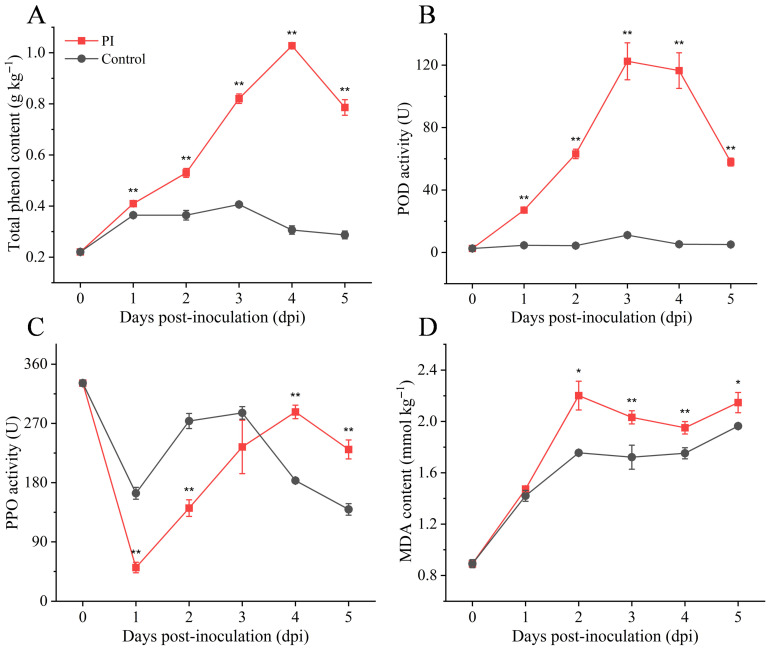
Defense responses and oxidative damage induced by *P. sclerotigenum* infection. (**A**) Total polyphenol content; (**B**) POD activity; (**C**) PPO activity; (**D**) MDA content. All values are expressed as mean ± standard deviation (*n* = 3). Analysis performed using Student’s *t*-test, *: *p* < 0.05, **: *p* < 0.01.

As shown in Figure 4, when infected by *P. sclerotigenum*, yams actively initiated a multi-tiered defense response. As key secondary defense metabolites, total phenolic content accumulated continuously, peaking on day 4 (Figure 4A). Defense-related enzyme activities were also rapidly mobilized. POD activity significantly increased on day 3 (Figure 4B). Conversely, PPO activity was suppressed at 1 dpi but subsequently strongly induced, even exceeding control levels in the late infection stage (Figure 4C). MDA, a marker of membrane lipid peroxidation, showed a significant increase in the PI group and remained consistently higher than the control group from the mid-infection stage onwards (Figure 4D). This indicated severe disruption of the cell membrane system in yams.

### 3.5. Global Metabolic Analysis Based on Metabolomics

**Figure 5:**
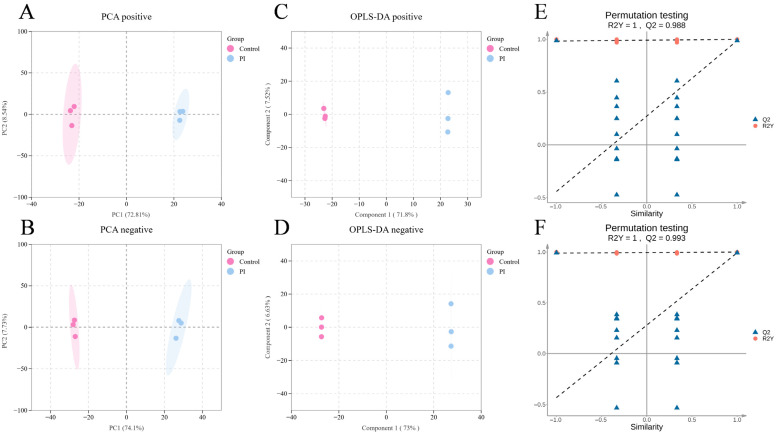
Global metabolic analysis based on non-targeted metabolomics. (**A**,**B**) PCA score plots in positive-ion mode and negative-ion mode; (**C**,**D**) OPLS-DA score plots in positive-ion mode and negative-ion mode; (**E**,**F**) Permutation test plots of OPLS-DA models in positive-ion mode and negative-ion mode.

Using non-targeted metabolomics, we systematically analyzed host metabolite changes at day 3 of *P. sclerotigenum* infection in yams. A total of 2091 metabolites were detected, including 865 identified in positive ion mode and 1226 in negative ion mode. Principal components analysis (PCA) and orthogonal partial least squares discriminant analysis (OPLS-DA) revealed complete separation of metabolic profiles between the control and PI groups. The models were validated via permutation tests, confirming significant and reliable metabolic differences between groups. This indicated that by day 3 of infection, *P. sclerotigenum* had induced substantial alterations in the metabolic profile of yams (Figure 5A–F).

### 3.6. Differentially Expressed Metabolite (DEM) Analysis

**Figure 6:**
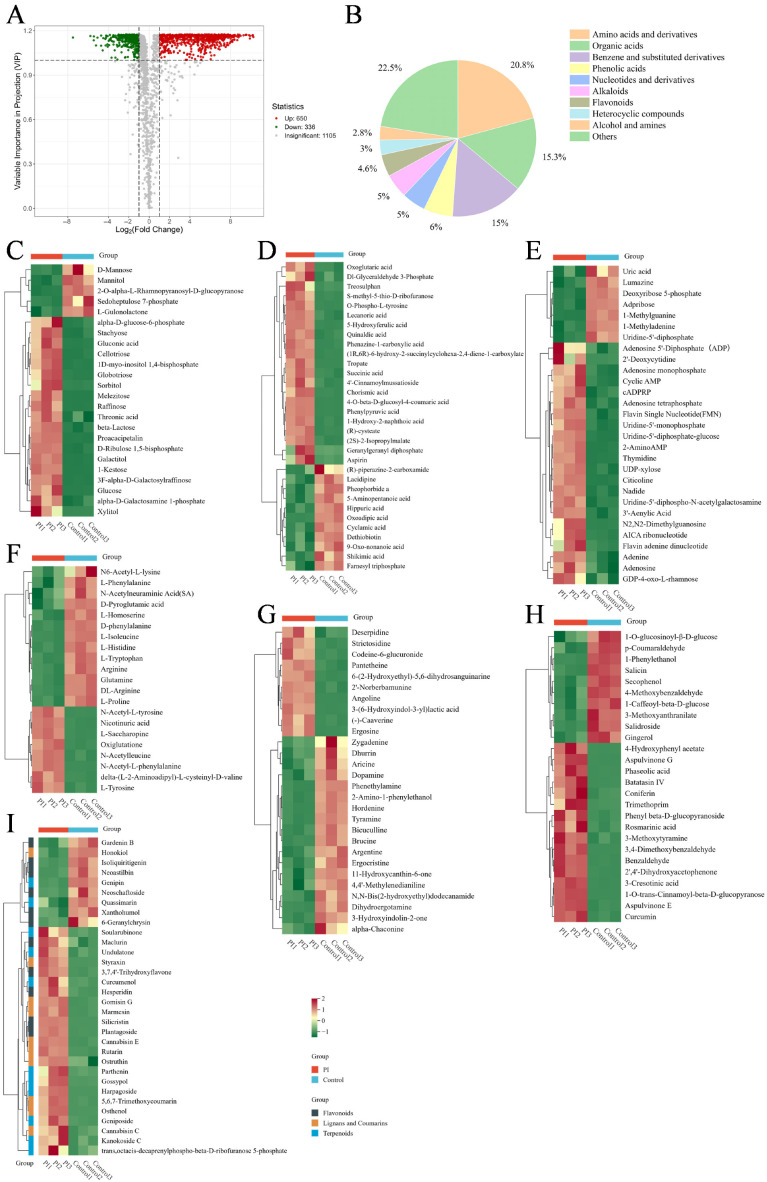
Analysis of DEMs in the PI group and the control group. (**A**) VIP volcano plot of DEMs; (**B**) Pie chart showing the proportion of DEMs by category; (**C**) Abundance clustering heatmap of sugar and sugar alcohol DEMs; (**D**) Abundance clustering heatmap of organic acid DEMs; (**E**) Abundance clustering heatmap of nucleotide and derivative DEMs; (**F**) Abundance clustering heatmap of amino acid and derivative DEMs; (**G**–**I**) Abundance clustering heatmaps of secondary metabolite DEMs.

Using variable importance in projection (VIP) > 1.0 and fold change (FC) ≥ 2.0 or ≤0.5 as screening criteria, we identified a total of 986 DEMs, comprising 650 upregulated DEMs and 336 downregulated DEMs (Figure 6A). These DEMs encompassed diverse biochemical categories, including 205 amino acids and their derivatives (20.79%), 151 organic acids (15.31%), 148 benzene and its derivatives (15.01%), 59 phenolic acids (5.98%), 49 nucleotides and their derivatives (4.97%), 49 alkaloids (4.97%), 45 flavonoids (4.56%), and other substances (Figure 6B). This indicated that the metabolic changes induced by *P. sclerotigenum* primarily concentrated on amino acids and organic acids. Among the 986 DEMs, 295 possessed detailed annotation information in the KEGG database. Therefore, we conducted further group analysis on categories with a larger number of substances. We focused on the change patterns in categories such as carbohydrates, amino acids and their derivatives, organic acids, nucleotides and their derivatives, as well as secondary metabolites including alkaloids, phenolic acids, flavonoids, terpenoids, lignans, and coumarins (Figure 6C–I). Detailed information on these substances is provided in Table S4.

Following infection, most sugar content increased. In the PI group, both cellulose degradation products, like Cellotriose, and glycolipid hydrolysis products from cell membranes (e.g., Globotriose) showed elevated levels. Several glycolysis-related metabolites were upregulated, including glucose and alpha-D-glucose-6-phosphate. Additionally, we observed elevated levels of highly hydrophilic sugar alcohols (e.g., sorbitol, galactitol, xylitol) in the PI group. Oxiglutathione, an oxidation product of glutathione, was up-regulated in the PI group. Over 60% of organic acids showed increased levels in the PI group. These included signaling molecules associated with protein phosphorylation (e.g., O-Phospho-L-tyrosine) and compounds with broad-spectrum antimicrobial activity (e.g., Lecanoric acid and 1-Hydroxy-2-naphthoic acid). We also identified upregulation of phenylpropanoid pathway-associated compounds, including 4-O-beta-D-glucosyl-4-coumaric acid, 5-hydroxyferulic acid, and 4′-cinnamoylmussatioside. Intermediates of glycolysis and the tricarboxylic acid cycle were also elevated, including Dl-glyceraldehyde 3-phosphate, succinic acid, and oxoglutaric acid. Furthermore, levels of numerous secondary metabolites—including phenolic acids, flavonoids, terpenoids, lignans, and coumarins—were significantly increased in the PI group.

### 3.7. Metabolic Pathway Enrichment Analysis

**Figure 7:**
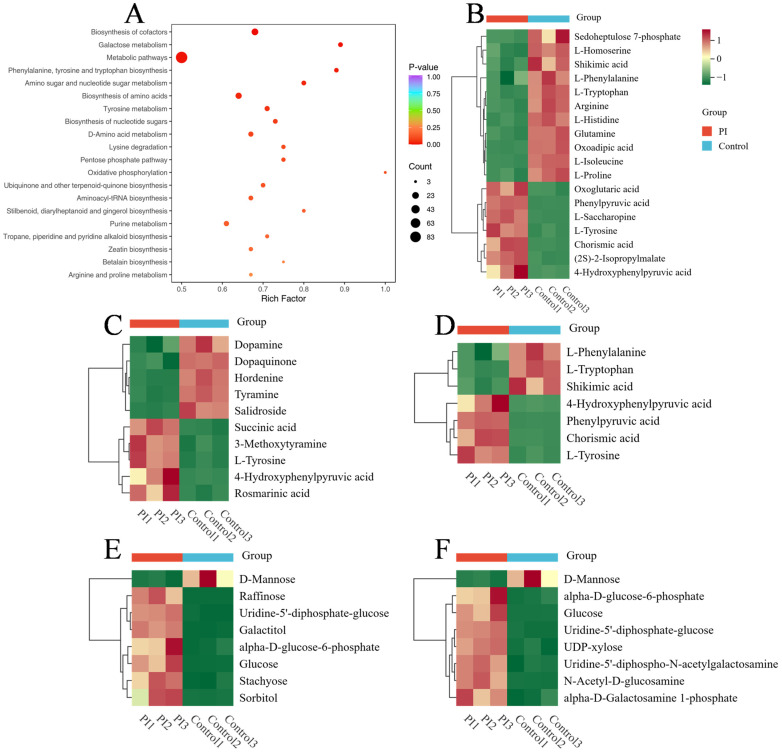
Metabolic pathway enrichment analysis and abundance clustering of key metabolites for DEMs. (**A**) Bubble plot of metabolic pathway enrichment analysis for DEMs between the PI group and the control group; (**B**) Heatmap of abundance clustering for DEMs in the amino acid biosynthesis pathway; (**C**) Heatmap of abundance clustering for DEMs in the biosynthesis pathways of phenylalanine, tyrosine, and tryptophan; (**D**) Abundance clustering heatmap of DEMs in the tyrosine metabolic pathway; (**E**) Abundance clustering heatmap of DEMs in the galactose metabolic pathway; (**F**) Abundance clustering heatmap of DEMs in the amino sugar and nucleotide sugar metabolic pathways.

Further pathway enrichment analysis of DEMs using the KEGG database revealed that 118 DEMs were annotated across 83 metabolic pathways. We selected the top 20 pathways with the smallest *p*-values and generated a pathway enrichment bubble plot (Figure 7A). Significantly enriched key metabolic pathways included cofactor biosynthesis, galactose metabolism, overall metabolic pathways, phenylalanine, tyrosine, and tryptophan biosynthesis, amino sugar and nucleotide sugar metabolism, amino acid biosynthesis, and tyrosine metabolism. Most of these enriched pathways were associated with carbohydrate metabolism and amino acid metabolism. Within amino acid metabolism pathways (e.g., tyrosine metabolism, phenylalanine, tyrosine, and tryptophan biosynthesis, and amino acid biosynthesis pathways), the co-enriched metabolites were L-Tyrosine and 4-Hydroxyphenylpyruvic acid, both of which were upregulated in the PI group. In carbohydrate metabolism pathways (e.g., galactose metabolism, amino sugar and nucleotide sugar metabolism), the co-enriched substances were uridine-5′-diphosphate-glucose, alpha-D-glucose-6-phosphate, D-mannose, and glucose. Except for D-mannose, the other three substances showed increased levels in the PI group. These co-enriched metabolites were considered core hubs in metabolic networks (Figure 7B–F).

## 4. Discussion

Fungal diseases cause significant economic losses in postharvest fruit and vegetables, particularly in developing countries, with loss rates reaching up to 45% [25]. *Penicillium* fungi, as common spoilage fungi, play a key role in the decay and rot of stored agricultural products such as apples and citrus, with their toxins posing significant threats to human health [26,27]. Research on post-harvest *Penicillium* disease in yams remains insufficient. Therefore, this study isolated and identified microorganisms from decayed Lichuan yams, yielding two bacterial strains and four fungal strains. All four isolated fungi were confirmed as pathogens causing postharvest decay in fruit and vegetables [28,29,30,31,32,33]. In vitro inoculation experiments validated *P. sclerotigenum* as the predominant pathogen responsible for postharvest decay in the tested Lichuan yam, and this fungus is also the causative agent of decay in Foshou yam [34].

During the early stages of *P. sclerotigenum* infection, processes such as spore germination and hyphal invasion occur, with infection symptoms gradually manifesting. By the mid-to-late stages of *P. sclerotigenum* infection, water loss in the yam increases sharply, and water activity continues to decline. This may result from *P. sclerotigenum* secreting cell wall-degrading enzymes such as pectinases and cellulases, which jointly disrupt the epidermis and parenchyma tissue structure of the yam [35]. Degradation of the cell wall not only weakens the physical barrier against water evaporation but may also compromise the structural integrity required to maintain turgor pressure, leading to the leakage of cellular contents. This provides a moist microenvironment conducive to pathogen colonization and spread [36]. Although reduced water activity typically inhibits microbial growth, *P. sclerotigenum* demonstrated strong pathogenicity in this study, suggesting it possessed effective osmotic regulation mechanisms that enable adaptation to—or even exploitation of—environmental changes associated with decreased water activity.

Research found that during the early stages of *P. sclerotigenum* infection, the starch and soluble sugar content in yams accumulate simultaneously. This likely represents a stress response by the plant to combat the pathogen. Rapid starch breakdown provides energy and carbon skeletons for energy-intensive processes such as defense-related gene expression, protein synthesis, and reactive oxygen species burst [37]. However, this adaptation may inadvertently supply readily available sugars to the pathogen. Similarly, the upregulation of protein content in the early infection stage may result from the extensive synthesis of disease-related proteins and defense enzymes [38]. Metabolomics data showing broadly elevated amino acid levels directly corroborate this. However, by the late infection stage, as host tissue necrosis progresses and pathogen populations surge, the accumulated carbon and nitrogen nutrients are rapidly depleted by the pathogen, leading to a subsequent decline in their levels. This dynamic shift reveals a metabolic competition between the yam’s defense mechanisms and the pathogen’s nutrient acquisition strategy, ultimately resulting in the exhaustion of host nutrient resources.

In response to *P. sclerotigenum* infection, yams activate a multi-tiered chemical defense system. The sustained accumulation of total phenolics, coupled with dynamic changes in POD and PPO activity, collectively form a defense system with both antioxidant and antibacterial functions [39]. Metabolomics data further support this: significant upregulation of key phenylpropanoid pathway compounds—5-hydroxyferulic acid, 4-O-beta-D-glucosyl-4-coumaric acid, and 4′-cinnamoylmussatioside—indicated this defense pathway’s activation and crucial role [40]. Notably, PPO activity was significantly suppressed during early infection (1 dpi), potentially representing a virulence strategy of *P. sclerotigenum* to weaken the yam’s ability to convert phenolic compounds into toxic quinones [41]. However, sustained pathogen pressure eventually overwhelmed the antioxidant defense capacity of the yam, leading to severe lipid peroxidation in cell membranes. Damage to the membrane system not only causes leakage of intracellular substances and disrupts metabolic compartmentalization but may also initiate programmed cell death, thereby accelerating tissue softening and decay [42].

Metabolomic analysis indicated that *P. sclerotigenum* infection exerted a comprehensive impact on yam metabolism, profoundly disrupting carbohydrate and amino acid pathways while linking to core primary metabolic processes such as glycolysis and the TCA cycle through key intermediates. Accumulation of alpha-D-glucose-6-phosphate not only provides a sugar donor for primary metabolism but also supplies energy for energy-intensive defense processes like cell wall synthesis and antimicrobial compound production [43,44]. This extensive metabolic reprogramming reflects the substantial metabolic cost incurred by the host in responding to biotic stress. Energy and carbon skeletons are redirected toward defense-related synthetic pathways, potentially compromising fundamental growth and maintenance functions [45].

**Figure 8:**
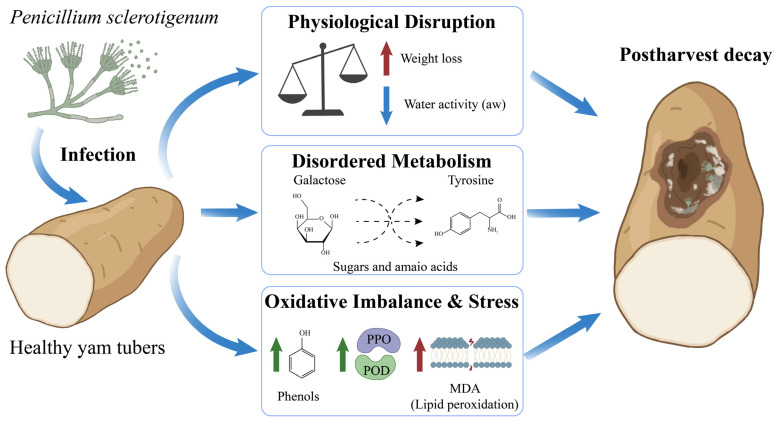
Simplified schematic diagram of the infection mechanism of *P. sclerotigenum* on yams.

In summary, we outlined a schematic diagram of the infection mechanism of *P. sclerotigenum* on postharvest yams (Figure 8). Despite initiating complex defense responses and metabolic reorganization, yam ultimately progresses toward tissue necrosis. This suggested that for yams entering the storage phase, their metabolic plasticity and resource reserve capacity proved insufficient to sustain defense against pathogen invasion during this highly energy-demanding metabolic competition.

## 5. Conclusions

In summary, we identified *P. sclerotigenum* as the dominant spoilage fungus in post-harvest decay of Lichuan yams and systematically elucidated its spoilage mechanisms at physiological, biochemical, and metabolomic levels. *P. sclerotigenum* induces postharvest decay by disrupting the water retention structures of yams and interfering with and exploiting their carbon and nitrogen metabolic networks. Although yams initiate comprehensive responses, including secondary metabolism and enzymatic defenses, sustained pathogen stress ultimately led to oxidative damage and systemic metabolic dysregulation. By integrating physiological phenotyping with metabolomics data, this study provides novel insights into the interaction mechanisms between yams and *Penicillium*. Future research should focus on key cell wall degradation enzymes and their regulatory mechanisms in *P. sclerotigenum*, while exploring the application potential of preemptively enhancing yams’ defense metabolic networks through exogenous signaling molecules to improve postharvest disease resistance.

## Data Availability

The original contributions presented in this study are included in the article/Supplementary Material. Further inquiries can be directed to the corresponding author.

## Supplementary Materials

The following supporting information can be downloaded at: https://www.mdpi.com/article/10.3390/jof12030225/s1, Table S1: Primer names and sequences used for microbial identification; Table S2: UPLC Conditions. (A = Water containing 0.1% Formic acid, B = Acetonitrile containing 0.1% Formic acid); Table S3: MS Conditions; Table S4: Detailed information on differential metabolites in healthy yam infected with *P. sclerotigenum* on day 3.

## Abbreviations

The following abbreviations are used in this manuscript:

POD	Peroxidase
PPO	Phenol oxidase
MDA	Malondialdehyde
DEMs	Differentially expressed metabolites
PCA	Principal components analysis
OPLS-DA	Orthogonal partial least squares discriminant analysis
